# Cancer‐associated fibroblast‐derived exosomal microRNA‐24‐3p enhances colon cancer cell resistance to MTX by down‐regulating CDX2/HEPH axis

**DOI:** 10.1111/jcmm.15765

**Published:** 2021-02-23

**Authors:** Hong‐Wei Zhang, Yi Shi, Ji‐Bin Liu, Hui‐Min Wang, Pei‐Yao Wang, Zhi‐Jun Wu, Liu Li, Li‐Peng Gu, Ping‐Sheng Cao, Gao‐Ren Wang, Yu‐Shui Ma, Da Fu

**Affiliations:** ^1^ Central Laboratory for Medical Research Shanghai Tenth People's Hospital Tongji University School of Medicine Shanghai China; ^2^ Cancer Institute Nantong Tumor Hospital Nantong China; ^3^ Institute of Interdisciplinary Integrative Biomedical Research Shanghai University of Traditional Chinese Medicine Shanghai China; ^4^ Department of Oncology Nantong Second People's Hospital Nantong China; ^5^ Department of Radiotherapy Nantong Tumor Hospital Nantong China

**Keywords:** cancer‐associated fibroblasts, caudal‐related homeobox 2, chemoresistance, colon cancer, hephaestin, methotrexate, microRNA‐24‐3p

## Abstract

MicroRNA‐24‐3p (miR‐24‐3p) has been implicated as a key promoter of chemotherapy resistance in numerous cancers. Meanwhile, cancer‐associated fibroblasts (CAFs) can secret exosomes to transfer miRNAs, which mediate tumour development. However, little is known regarding the molecular mechanism of CAF‐derived exosomal miR‐24‐3p in colon cancer (CC). Hence, this study intended to characterize the functional relevance of CAF‐derived exosomal miR‐24‐3p in CC cell resistance to methotrexate (MTX). We identified differentially expressed HEPH, CDX2 and miR‐24‐3p in CC through bioinformatics analyses, and validated their expression in CC tissues and cells. The relationship among HEPH, CDX2 and miR‐24‐3p was verified using ChIP and dual‐luciferase reporter gene assays. Exosomes were isolated from miR‐24‐3p inhibitor–treated CAFs (CAFs‐exo/miR‐24‐3p inhibitor), which were used in combination with gain‐of‐function and loss‐of‐function experiments and MTX treatment. CCK‐8, flow cytometry and colony formation assays were conducted to determine cell viability, apoptosis and colony formation, respectively. Based on the findings, CC tissues and cells presented with high expression of miR‐24‐3p and low expression of HEPH and CDX2. CDX2 was a target gene of miR‐24‐3p and could up‐regulate HEPH. Under MTX treatment, overexpressed CDX2 or HEPH and down‐regulated miR‐24‐3p reduced cell viability and colony formation and elevated cell apoptosis. Furthermore, miR‐24‐3p was transferred into CC cells via CAF‐derived exosomes. CAF‐derived exosomal miR‐24‐3p inhibitor diminished cell viability and colony formation and increased cell apoptosis in vitro and inhibited tumour growth in vivo under MTX treatment. Altogether, CAF‐derived exosomal miR‐24‐3p accelerated resistance of CC cells to MTX by down‐regulating CDX2/HEPH axis.

## 1 INTRODUCTION

Colon cancer (CC) represents the 2nd most common reason for cancer‐related death, which arises from a local adenoma detected in the intestinal epithelium to an aggressive malignant tumour and might potentially metastasize to the liver.^1^ Currently, the therapeutic regimens for CC include chemotherapy.^2^ Methotrexate (MTX), a well‐known antitumour agent, is a folic acid analogue with high cytotoxicity,^3^ which is widely used as a standard chemotherapy for a wide array of cancers such as CC ^4^ and colorectal cancer (CRC).^5^ However, resistance to chemotherapy that develops in tumour cells limits their therapeutic efficacy in patients.^6^ Moreover, chemotherapy is not the only available treatment, and target therapy has been demonstrated to better the survival and quality of life of cancer patients life.^7^, ^8^

Cancer‐associated fibroblasts (CAFs), amongst the most abundant cell type occurringin tumour matrix, play a pivotal role in context of the development of cancers, including breast cancer, pancreatic cancer and colon cancer.^9^ It is reported that CAFs can secret exosomes to regulate tumour progression.^10^ Exosomes belong to extracellular vesicles and can function as non‐invasive biomarkers for cancer treatment^11^ influencing drug resistance.^12^ Currently, the transfer of microRNAs (miRNAs) from fibroblasts *via* microvesicles to tumour cells is a promising approach in the cessation of chemoresistance of tumour cells.^13^ Furthermore, CAF‐derived exosome carrying miRNAs contributes to chemoresistance of various cancers, including pancreatic cancer and head and neck cancer.^14^, ^15^

miRNAs participate in numerous cellular processes, whose dysregulation has vital functionality in the context of the development and progression of cancers.^16^ At present, mounting evidence has revealed that miRNAs promote chemotherapy resistance of CC cells.^17^, ^18^, ^19^ Furthermore, microRNA‐24‐3p (miR‐24‐3p) has been documented to regulate chemoresistance in several cancers such as in breast cancer^20^ and ovarian cancer.^21^ Moreover, a study previously confirmed that CAF‐derived exosome carrying lncRNA H19 plays a role in the enhancement of CRC cell chemoresistance.^22^ Furthermore, microarray data of our study predicted that miR‐24‐3p were capable of targeting caudal‐related homeobox 2 (CDX2) in CC. Moreover, another study showed that CDX2 directly regulates the transcription of hephaestin (HEPH).^23^ With the aforementioned preliminary predictions and previous studies considered as the basis, we hypothesized that exosomal miR‐24‐3p derived from CAFs played a role in CC resistance to MTX *via* CDX2/HEPH axis and conducted a study, including the co‐culturing of exosomes isolated from CAFs with CC cells to test this hypothesis.

## 2 METHODS AND MATERIALS

### 2.1 Ethical statement

The study was conducted with the approval of the Ethics Committee of Shanghai Tenth People's Hospital, Tongji University School of Medicine. All of the participants or their caregivers signed informed consent prior to the enrolment in this study. All animal studies were undertaken in accordance with the guidelines issued in the Guide for the Care and Use of Laboratory Animals of the National Institutes of Health.

### 2.2 Microarray‐based gene expression analysis

MTX‐resistant CC‐related profile GSE11440 was retrieved from the Gene Expression Omnibus (GEO) database available at (https://www.ncbi.nlm.nih.gov/geo). GSE11440 contained 3 drug‐sensitive samples and 3 drug‐resistant samples. Differentially expressed genes (DEGs) were analysed and selected using limma package.^24^ DEGs were identified with |log2 fold change (FC)| > 2.0 and FDR < 0.05 as threshold. The expression of CDX2 and HEPH in CC of Cancer Genome Atlas (TCGA) database analysis was obtained from the UALCAN database available at **(** http://ualcan.path.uab.edu/analysis.html). miRNA microarray data in fibroblast microvesicles were downloaded from EVmiRNA database (http://bioinfo.life.hust.edu.cn/EVmiRNA). The mirDIP database acquired from (http://ophid.utoronto.ca/mirDIP/index.jsp#r) and TargetScan database acquired from (http://www.targetscan.org/vert_71/) were used to predict miRNAs regulating CDX2. The expression of miR‐24‐3p in CC of TCGA database was searched in the starBase database (starbase.sysu.edu.cn).

### 2.3 Study subjects

Colon cancer tissues and adjacent normal tissues were acquired from 28 patients with stage I‐III CC who received surgical treatment at Shanghai Tenth People's Hospital, Tongji University School of Medicine, from January 2015 to March 2019. The enrolled patients included 16 males and 12 females, aged 55‐69 years showing a mean age of 62.04 ± 4.26 years. Prior to surgery, the complete medical history was obtained from all 28 patients and each patient received a physical examination.

### 2.4 Cell culture

Normal human colonic epithelial cells NCM‐460 and CC cell lines including SW620, HT29, LoVo and SW1116 were purchased from the American Type Culture Collection (ATCC; Manassas, VA, USA). All cells were grown in Ham's F12 medium (GIBCO, Barcelona, Spain) which was supplemented with 7% foetal bovine serum (FBS) under humidified environment at the controlled temperature of 37°C and atmosphere of 5% CO_2_.

The CC tissues were minced and re‐suspended in a Dulbecco's modified eagle's medium (DMEM) containing penicillin‐streptomycin (Invitrogen, Carlsbad, CA, USA), amphotericin B (Invitrogen, Carlsbad, CA, USA), 3% collagenase and 20% FBS (Gibco, Grand Island, NY, USA) for 2 hours. The sample was filtered using an 8‐μm filter for 2 hours for the removal of undigested debris. Single‐cell suspension that was supplemented with viable fibroblasts was plated in 24‐well plates in DMEM containing 10% FBS for duration of 2‐3 weeks and then transferred to T75 flasks for further culture. The tenth passage of fibroblasts was used for subsequent experiments. Normal fibroblasts (NFs) were obtained 10 cm from the edge of the tumour infiltration. The morphology of isolated CAFs and NFs was observed under an inverted microscope. CAFs and NFs were identified through the determination of expression of α‐smooth muscle actin (α‐SMA), fibroblast activation protein (FAP), fibroblast‐specific protein 1 (FSP‐1) and vimentin using immunofluorescence and Western blot analysis.

### 2.5 Cell transfection

The cells were subjected to seeding into 6‐well plates containing 1 mL Ham's F12 medium before transfection. After 18 hours, expression plasmids pCMV6‐XL5‐CDX2 (CDX2) and pCMV6‐XL5‐HEPH (HEPH) (OriGene Technologies, Rockville, MD, USA) and small interfering RNAs (siRNA) targeting CDX2 (si‐CDX2) and HEPH (si‐HEPH) were used for transfection, which was conducted according to the manufacturer's instructions of Lipofectamine^TM^ 2000 (Invitrogen, Carlsbad, CA, USA). In order to monitor the overexpression of CDX2 and HEPH, mRNA level or protein level was measured after 24 or 48 hours, respectively. miR‐24‐3p mimic, inhibitor and their controls were produced by GENECHEM (Shanghai, China) and transfected into cells using Lipofectamine 2000. MTX (Sigma, Sigma‐Aldrich, St. Louis, MO, USA) was added to cells after 24‐h transfection to analyse the sensitivity of the cells to MTX. The cell viability was assessed by cell counting kit (CCK)‐8 assay. Empty vector (pCMV6‐XL5) and si‐negative control (NC) were used as NC.

### 2.6 Reverse transcription‐quantitative polymerase chain reaction (RT‐qPCR)

Total RNA content acquired from tissues or cells was extracted by means of TRIzol (Invitrogen, Carlsbad, NY, USA). Reverse transcription was performed to detect mRNA and miRNA expressions in accordance with the instructions of cDNA Synthesis Kit (RR047A, TaKaRa, Tokyo, Japan) and miRNA First Strand cDNA Synthesis (Tailing Reaction) Kit (B532451‐0020, Shanghai Sangon Biotech Company, Shanghai, China), respectively, to generate cDNA. RT‐qPCR was conducted using iTaq™ Universal SYBR^®^ Green Supermix Kit (Bio‐Rad, Hercules, CA, USA). The mRNA levels were normalized to glyceraldehyde 3‐phosphate dehydrogenase (GAPDH), and the miRNA level was normalized to U6. miRNA in culture medium or exosomes were extracted from fixed volume (350 µL) by mirVana PARIS Kit (Ambion, Austin, TX, USA). The miRNA levels in culture medium and exosomes were normalized to U6. The synthesis of primers shown in Table 1 was conducted by Aoke Biotechnology Co., Ltd (Beijing, China). Relative quantitative results were processed by means of 2^−ΔΔCt^ method.

**Table 1:** Primer sequences for RT‐qPCR

Gene	Primer sequence
miR‐24‐3p	F: 5′‐TGGCTCAGTTCAGCAGGAACAG‐3′
	F: 5′‐ACCGAGTCAAGTCGTCCTTGTC‐3′
U6	F: 5′‐CGAGCACAGAATCGCTTCA‐3′
	F: 5′‐CTCGCTTCGGCAGCACATAT‐3′
HEPH	F: 5′‐TCCAATCGAATGCATGCATGCT‐3′
	R: 5′‐AACATAACCCATGTACA‐3′
CDX2	F: 5′‐GATGATACTGCTGGGTACTG‐3′
	R: 5′‐TCTCCTTTGCTCTGCGGTTC‐3′
GAPDH	F: 5′‐GCACCGTCAAGGCTGAGAAC‐3′
	R: 5′‐TGGTGAAGACGCCAGTGGA‐3′

Abbreviations: CDX2, caudal‐related homeobox 2; F, forward; GAPDH, glyceraldehyde‐3‐phosphate dehydrogenase; HEPH, hephaestin; miR‐24‐3p, microRNA‐24‐3p; R, reverse; RT‐qPCR, reverse transcription‐quantitative polymerase chain reaction.

### 2.7 Western blot analysis

Total protein content was extracted using lysis buffer that contained (1%NP‐40, 20 mmol/L Tris, pH8.0, 137 mmol/L NaCl, 0.5 mmol/L EDTA, 0.1% sodium dodecyl sulphate [SDS], 10% glycerol, 10 mmol/L Na2P2O7, 10 mmol/L NaF, 1 μg/mL aprotinin, 10 μg/mL leupeptin, 1 mmol/L sodium vanadate and 1 mmol/L PMSF). The protein concentration was estimated using bicinchoninic acid (BCA) protein assay (BCA1, Sigma‐Aldrich, St. Louis, MO, USA). The isolated proteins were separated by SDS‐polyacrylamide gel electrophoresis, and then transferred to polyvinylidene fluoride membrane. The membrane was probed with addition of primary anti‐rabbit antibodies against HEPH (1:200, ab108003), CDX2 (1:10 000, ab76541), GAPDH (1:2500, ab9485), α‐SMA (1:1000, ab32575), FAP (1:1000, ab53066), FSP‐1 (1:1000, ab124805), vimentin (1:1000, ab193555), CD63 (1:1000, ab134045), CD81 (1:1000, ab109201) and tumour susceptibility gene 101 (TSG101; 1:5000, ab125011) at the controlled temperature of 4°C overnight prior to re‐probing with secondary antibody. All of the preceding antibodies were acquired from Abcam (Cambridge, UK). The image was subjected to greyscale analysis using Pierce ECL Western Blotting Substrate (Thermo Fisher Scientific, Waltham, MA, USA). Image density of the immunoblotting was subjected to determination with the application of Gel densitometry (Bio‐Rad, Hercules, CA, USA).

### 2.8 Chromatin immunoprecipitation (ChIP) assay

ChIP was carried out by means of the ChIP assay kit (Beyotime Institute of Biotechnology, Shanghai, China) per manufacturer's instructions. Briefly, CC cells were subjected to cross‐linked with 1% formaldehyde solution for 10 minutes at room temperature and quenched with 125 nmol/L glycine. DNA was sheared to an average size of 200‐150 bp by ultra‐sonication. The lysis buffer was then immunoprecipitated with anti‐CDX2 (clone 7C7/D4, Bio‐Genex Laboratories Inc, San Ramon, CA, USA) or immunoglobulin G antibody (Catalog No: 61112, Active Motif Offices, Active Motif, Carlsbad, CA, USA). The immunoprecipitated DNA fragment was analysed by RT‐qPCR assay. The primer sequence was as follows: F: 5′‐TCCTCGTCTCTCCTTCTTGC‐3′, R: 5′‐AGAAGGTCAGGGCTGAGACTC‐3′.

### 2.9 Dual‐luciferase reporter gene assay

CDX2‐3′‐untranslated region (3′UTR) was subjected to artificial synthesis and insertion into psiCHECK‐2 vector (Promega, Madison, WI, USA). Mutant (MUT) of complementary sequence of seed sequences was designed on the basis of CDX2 wild‐type (WT) sequence and inserted into psiCHECK‐2 vector. Two recombinant plasmids, namely CDX2‐WT and CDX2‐MUT, were co‐transfected into human embryonic kidney 293T (HEK‐293T) cells with miR‐24‐3p mimic and mimic NC. Subsequent to a 48‐hours transfection, the cells were lysed. Following this, luciferase activity was measured using Glomax 20/20 luminometer (Promega, Madison, WI, USA). The luciferase activity was directly measured by the ratio of firefly luciferase activity to Renilla luciferase activity.

### 2.10 CCK‐8 assay

A total of 2000 HT29 or SW620 cells were seeded in 96‐well plates for duration of 12 hours prior to treatment of different concentrations of MTX (0, 20, 40, 60 or 80 nmol/L) for 5 d. Cell proliferation was detected using CCK‐8 (Dojindo Molecular Technologies, Kyushu, Japan). Each culture dish was added with 10 μL CCK‐8 solution for 2‐hours culture at 37°C. Cell viability was determined by absorbance (A) at wavelength of 450 nm (Wellscan MK3; Labsystems, Helsinki, Finland). Three replicate wells were set for each sample.

### 2.11 Clone formation assay

Transfected cells were subjected to seeding into 6‐well plates at 500 cells/well for the purpose of overnight incubation. The following day, cells were treated with 50 nmol/L MTX for duration of 24 hours. Then, the medium was replaced with MTX‐free medium. After 14 d, the cells were subjected to fixation by means of 4% paraformaldehyde, staining by means of 0.1% crystal violet and counting visually. Three replicate wells were set for each sample.

### 2.12 Flow cytometry

Total 60,000 cells were cultured in 1 mL medium for duration of 18 hours, followed by transfection. After duration of 24 hours, 50 nmol/L MTX was added into cells. Apoptosis was measured after 6 hours of MTX treatment. Under dark conditions, 5 μg/mL Annexin V‐fluorescein isothiocyanate (FITC) and 5 μg/mL propidium iodide (PI) (Sigma‐Aldrich, Madrid, Spain) were applied to stain cells for 15 minutes. A flow cytometer (Becton‐Dickinson, Franklin Lakes, NJ, USA) was adopted with the aim to examine cell apoptosis.

### 2.13 Isolation of exosomes

In order to isolate exosomes from the culture medium, CAFs or NFs were cultured for duration of 48 hours in DMEM/F12 medium which was supplemented with 10% FBS (exosome removal). The supernatant was collected, after which centrifugation was carried out at 300 × g for 10 minutes, at 2000 × *g* for 15 minutes and at 12 000 × *g* for 30 minutes to remove floating cells and cell debris from the culture supernatant. The supernatant was then passed through a 0.22‐μm filter (Millipore, Billerica, MA, USA). The supernatant obtained was passed through a membrane by centrifugation in an ultrafiltration apparatus (UFC900396, Millipore, Billerica, MA, USA) at 4 × 10^3^ × g for 1 hours. Finally, Exo Quick TC™ exosome separation reagent (EXOTC50A‐1, System Biosciences, Mountain View, CA, USA) was added to the concentrated solution of the supernatant at a ratio of 1:5. Exosomes were extracted based on the instructions provided on the kit (SBI, System Biosciences, Mountain View, CA, USA). The total protein concentration of the isolated exosomes was determined using a BCA protein assay kit (KeyGEN, Nanjing, Jiangsu, China).

### 2.14 Identification of exosomes

The morphology of the exosomes was observed by means of a transmission electron microscopy (TEM), FEI Tecnai 12, Philips, Eindhoven, the Netherlands. Briefly, exosomes were fixed on a copper grid with 4% paraformaldehyde. The copper grid was dried at room temperature for 10 minutes. The sample was subjected to staining with 2% uranyl acetate, drying for duration of 10 minutes, followed by observation at 100 KV. The size distribution of exosomes was analysed by measuring the Brownian motion rate using a Nanosight LM20 system equipped with fast video capture and particle tracking software (Nanosight, Amesbury, UK). Exosomal markers CD63, CD81 and TSG101 were determined by Western blot analysis.

### 2.15 Analysis of exosome internalization and miR‐24‐3p transfer

Total 5 × 10^5^ cells were re‐suspended in 500 μL exosome‐depleted medium, and incubation was carried out with 2 μg exosomes labelled by PKH‐67 at 37°C. The sample was transferred into ice and underwent 3 washes by means of cold PBS (pH = 7.4). Cells were kept in ice, and fluorescence measurement of living cells was performed with the use of a Leica TCS STED confocal microscope (Leica Microsystems, Wetzlar, Germany).

In Transwell co‐culture experiments, CAF cells were cultured on a Transwell (Thermo Fisher Scientific, Waltham, MA, USA) with 0.4‐mm aperture and transfected with miR‐24‐3p oligo (Biomics Biotechnology Corp, Nantong, China) labelled by 10 nmol/L red fluorescent Cy3 for 24 hours. The cells were then co‐cultured with HT29 cells or SW620 cells on coverslips at the bottom of Transwell. Subsequent to 24 hours, the cells were fixed by means of 4% paraformaldehyde and observed by means of a laser scanning confocal microscopy (LSCM).

### 2.16 Immunofluorescence assay

Cells were incubated on uncoated glass coverslips for duration of 24 hours, and then fixed by means of 4% paraformaldehyde for duration of 15 minutes at room temperature. The adherent cells were permeated in PBS containing 0.1% Triton X‐100, followed by blocking with 1% bovine serum albumin (BSA)/PBS containing 10% normal goat serum for 1 hours and incubation overnight with primary anti‐rabbit antibodies (Abcam, Cambridge, UK) against α‐SMA (1:500, ab32575), FAP (1:500, ab53066), FSP‐1 (1:250, ab124805) and vimentin (1:500, ab193555) at 4°C. Afterwards, cells were incubated with Alexa Fluor^®^ 647‐conjugated secondary donkey anti‐rabbit immunoglobulin G (IgG) antibody (ab150075, 1:500) for 1 hours. Finally, cells were fixed with Vectashield containing 4′6‐diamidino‐2‐phenylindole (DAPI) and observed under a LSCM.

### 2.17 Tumour xenografts in nude mice

Eighty BALB/c nude mice (at the age of 5  weeks, and weighing 12‐18 g) were attained from Hunan SJA Laboratory Animal Co., Ltd (Changsha, Hunan, China). HT29 cells received treatment with mimic NC, miR‐24‐3p mimic and CDX2. Total 5 × 10^6^ stably transfected HT29 cells were subjected to re‐suspension in PBS and subcutaneous injection subcutaneously into the flank of the mice to establish xenografts. On 16th day, MTX (10 mg/kg) or the same volume of 0.9% saline was injected intraperitoneally every 3 days when the volume of the xenograft reached 80‐200 mm^3^. To investigate the role of exosome‐derived miR‐24‐3p in the treatment of MTX, 10 μg exosomes from NFs (NFs‐exo), exosomes from CAFs (CAFs‐exo) or exosomes from CAFs transfected miR‐24‐3p inhibitor (CAFs‐exo/miR‐24‐3p inhibitor) were injected into the subcutaneous tumour every 3 days. The mice were killed on the 40th day following injection of HT29 cells into mice to obtain xenografts. Tumour volume (V) was calculated using the formula V = length × width^2^/2. The apoptosis in transplanted tumours was detected with the use of a TUNEL kit (Roche, Basel, Switzerland).

### 2.18 Statistical analysis

Statistical analysis for all data in the current study was implemented by means of SPSS 21.0 software (IBM Corp., Armonk, NY, USA). Measurement data were displayed as a form of mean ± standard deviation. The difference analysis between two groups was implemented by means of paired *t*test, and the difference analysis among multiple groups was implemented by means of anova with Tukey’s post hoc test. Data that were at different time‐points were compared by repeated‐measures anova, followed by Tukey's post hoc test. Pearson's test was used for correlation analysis. *P* < 0.05 was suggestive of statistically significant.

## 3 RESULTS

### 3.1 CDX2 and HEPH are down‐regulated in CC tissues and cells

**Figure 1:**
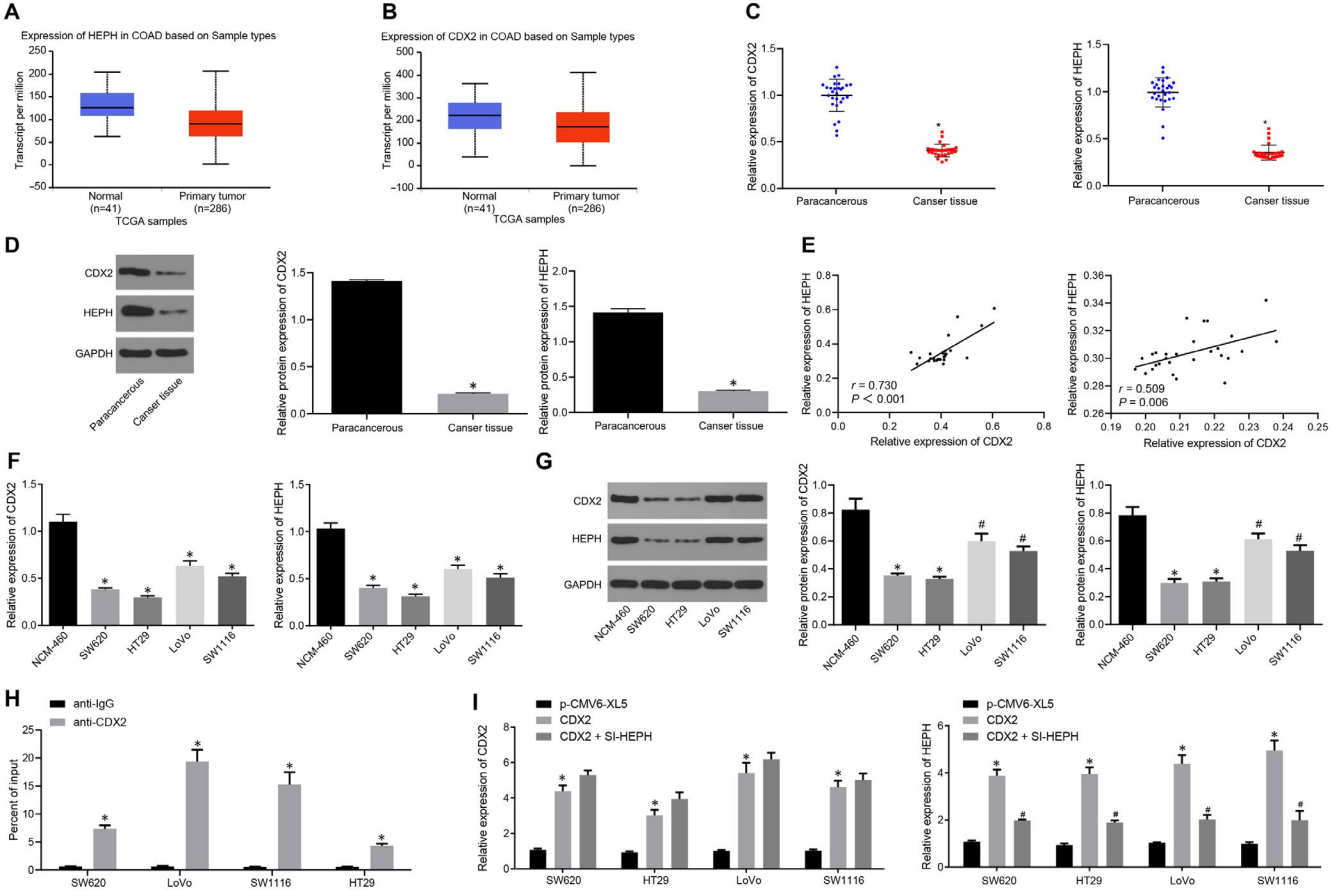
CDX2 and HEPH are poorly expressed in CC tissues and cells. A and B, HEPH and CDX2 expression in colon adenocarcinoma (COAD) samples of TCGA database, respectively. The red box plot represents the tumour samples, and the blue box plot represents the normal samples. C and D, CDX2 and HEPH expression in clinical samples of CC tissues and adjacent tissues determined by RT‐qPCR and Western blot analysis normalized to GAPDH (n = 28). E, Analysis correlation of mRNA (left) and protein (right) expression between CDX2 and HEPH in clinical samples of CC using the Spearman correlation test. F and G, CDX2 and HEPH expression in NCM‐460 cells and 4 CC cells (SW620, HT29, LoVo, SW1116) measured by RT‐qPCR and Western blot analysis normalized to GAPDH. H, The binding relationship between CDX2 and HEPH verified by ChIP experiment. I, CDX2 and HEPH expression in CC cells detected by RT‐qPCR after CC cells (SW620, HT29, LoVo and SW1116) was transfected with p‐CMV6‐XL5 empty vector, p‐CMV6‐XL5‐CDX2 (CDX2), or p‐CMV6‐XL5‐CDX2 + Si‐HEPH (CDX2 + si‐HEPH) normalized to GAPDH. ^*^ *P* < .05 vs adjacent tissues, NCM‐460 cells, anti‐IgG or CC cells transfected with pCMV6‐XL5. ^#^ *P* < .05 vs CC cells transfected with CDX2. Measurement data were expressed as mean ± standard deviation. Data of clinical samples were compared using paired *t* test, and data between two groups were analysed by unpaired *t* test. Comparisons among multiple groups were conducted by one‐way analysis of variance (ANOVA), followed by Bonferroni's post hoc test. Each cell experiment was repeated three times

Firstly, the correlation of CC with CDX2 and HEPH was explored. After analysing CC‐related differentially expressed gene in the TCGA database, it was revealed that expression of HEPH and CDX2 was markedly down‐regulated in CC samples compared with normal samples (Figure 1A,B). Furthermore, RT‐qPCR and Western blot analyses were adopted to examine CDX2 and HEPH expression in clinical samples of CC tissues and adjacent tissues. The results showed that CDX2 and HEPH expression in CC tissues was noticeably lower than that in adjacent tissues (Figure 1C,D). At the same time, it was found that there was a positive correlation of mRNA and protein expression between CDX2 and HEPH in clinical samples of CC (Figure 1E). CDX2 and HEPH expression in normal colonic epithelial cells (NCM‐460) and CC cells (SW620, HT29, LoVo and SW1116) was detected by RT‐qPCR and Western blot analyses, which displayed that CDX2 and HEPH expression was lower in CC cells than in NCM‐460 cells (Figure 1F,G), which is consistent with the results from clinical samples. Furthermore, ChIP experiment revealed that CDX2 and HEPH bound to each other (Figure 1H). Moreover, CC cells (SW620, HT29, LoVo and SW1116) were transfected with p‐CMV6‐XL5 empty vector, p‐CMV6‐XL5‐CDX2 (CDX2), or p‐CMV6‐XL5‐CDX2 + Si‐HEPH (CDX2 + si‐HEPH) and RT‐qPCR was adopted to determine the CDX2 and HEPH expression in CC cells, which revealed significantly increased CDX2 expression following transfection with p‐CMV6‐XL5‐CDX2, compared with empty vector. After cells were transfected with si‐HEPH, a marked reduction in HEPH expression was observed in comparison with cells transfected with p‐CMV6‐XL5‐CDX2 (CDX2) (Figure 1I). Further, we verified the efficiency of transfection and provided the original data under a fluorescence microscope in Figure S1. The aforementioned results indicated that CDX2 and HEPH were down‐regulated in CC and they were positively correlated in CC cells. CDX2 and HEPH were able to bind to each other, and CDX2 could increase HEPH expression.

### 3.2 Overexpression of CDX2 elevates HEPH expression to reduce CC cell resistance to MTX

**Figure 2:**
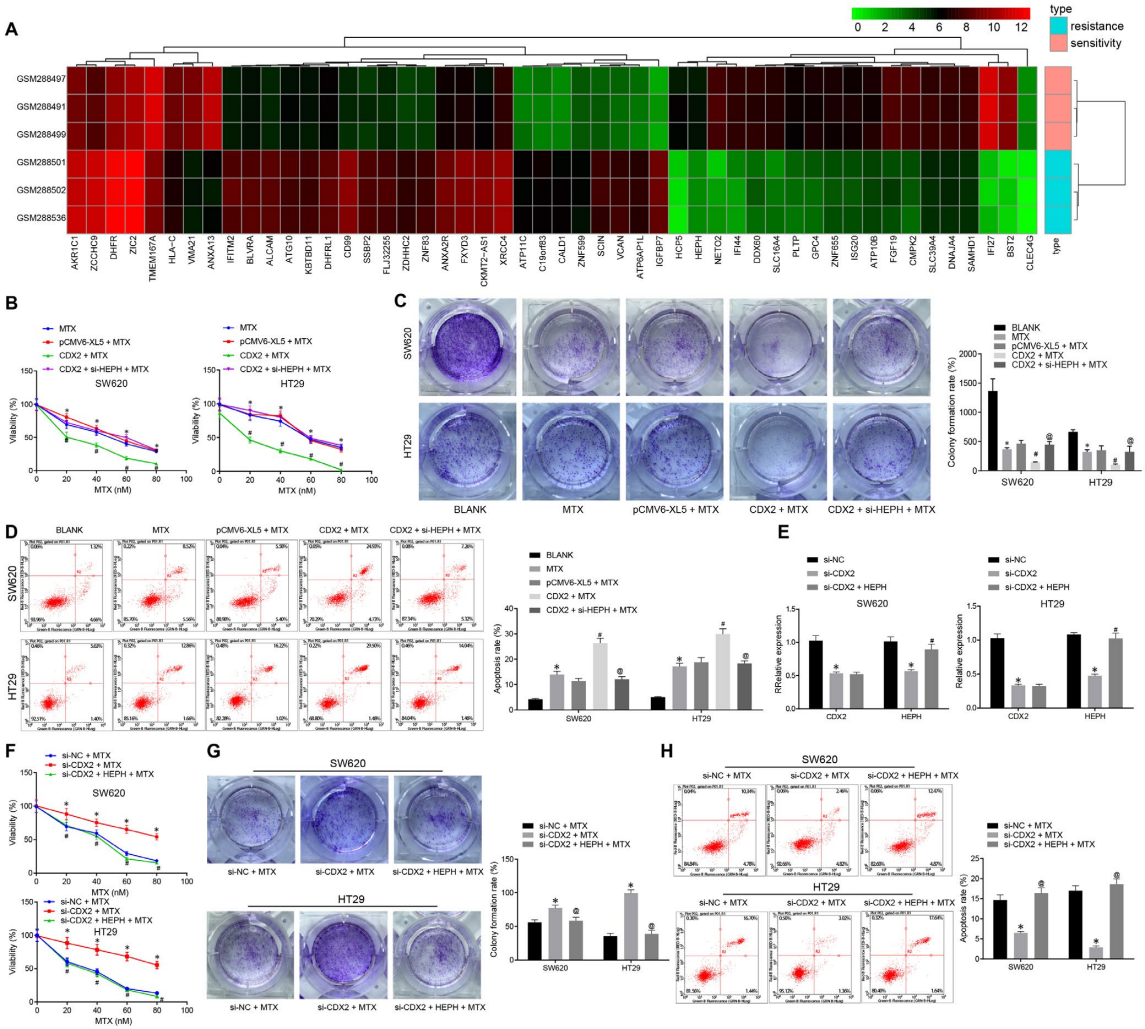
CDX2 overexpression up‐regulates HEPH to repress CC cell resistance to MTX. A, Heat map of differentially expressed genes in MTX‐resistant CC expression profile GSE11440. The abscissa refers to the sample number, the ordinate refers to the gene name, the left tree indicates the gene expression cluster, and the upper tree indicates the sample cluster. The upper right histogram was colour gradation, and each square in the FIGURE represents the expression of a gene in a sample. B, Cell viability of SW620 and HT29 cells transfected with CDX2 and si‐HEPH after 5 d of MTX treatment detected by CCK‐8 assay. C, Colony formation of MTX‐treated SW620 and HT29 cells after transfection with CDX2 and si‐HEPH assessed by colony formation assay. D, Apoptosis of MTX‐treated SW620 and HT29 cells after transfection with CDX2 and si‐HEPH examined by flow cytometry assay. E, CDX2 and HEPH expression in MTX‐treated SW620 and HT29 cells after transfection with si‐CDX2 and HEPH measured by RT‐qPCR normalized to GAPDH. F, Cell viability of SW620 and HT29 cells transfected with CDX2 and si‐HEPH after 5 d of MTX treatment detected by CCK‐8 assay. G, Colony formation of MTX‐treated SW620 and HT29 cells after transfection with si‐CDX2 and HEPH assessed by colony formation assay. H, Apoptosis of MTX‐treated SW620 and HT29 cells after transfection with si‐CDX2 and HEPH analysed by flow cytometry. ^*^ *P* < .05 vs. SW620 and HT29 cells without any treatment or MTX‐treated SW620 and HT29 cells. ^#^ *P* < .05 vs. MTX‐treated SW620 and HT29 cells transfected with pCMV6‐XL5. ^@^ *P* < .05 vs. MTX‐treated SW620 and HT29 cells transfected with CDX2 or si‐CDX2. Measurement data were expressed as mean ± standard deviation. Comparisons among multiple groups were conducted by one‐way analysis of variance (ANOVA). Data at different time‐points were compared by repeated‐measures ANOVA, followed by Bonferroni's post hoc test. Each cell experiment was repeated three times

To investigate the effect of CDX2 and HEPH on CC cell resistance to MTX, we first obtained the MTX‐resistant CC expression profile GSE11440 on the GEO database. It was found that the expression of HEPH in the MTX‐resistant samples was significantly reduced, and the expression change multiples and the significant *p* values ranked relatively high (Figure 2A). Subsequently, 2 CC cell lines SW620 and HT29 with the lowest expression of CDX2 and HEPH were selected for subsequent experiments to further explore the function of CDX2 and HEPH. CCK‐8 assay, colony formation assay and flow cytometry were conducted, and the results showed that MTX‐treated SW620 and HT29 cells exhibited decreased cell viability and colony formation and increased cell apoptosis. After MTX treatment, SW620 and HT29 cell viability and colony formation were reduced secondary to overexpressed CDX2, which was reversed by HEPH silencing (Figure 2B‐D). CDX2 was silenced, and HEPH was overexpressed in SW620 and HT29 cells (Figure 2E). In MTX‐treated SW620 and HT29 cells, cell viability and colony formation were strikingly elevated, and the apoptotic rate was remarkably diminished by si‐CDX2 treatment, which was abrogated by HEPH treatment (Figure 2F‐H). These findings suggested that after cells exposed to MTX, overexpression of CDX2 up‐regulated HEPH to reduce the resistance of CC cells to MTX.

### 3.3 CDX2 is the target gene of miR‐24‐3p

**Figure 3:**
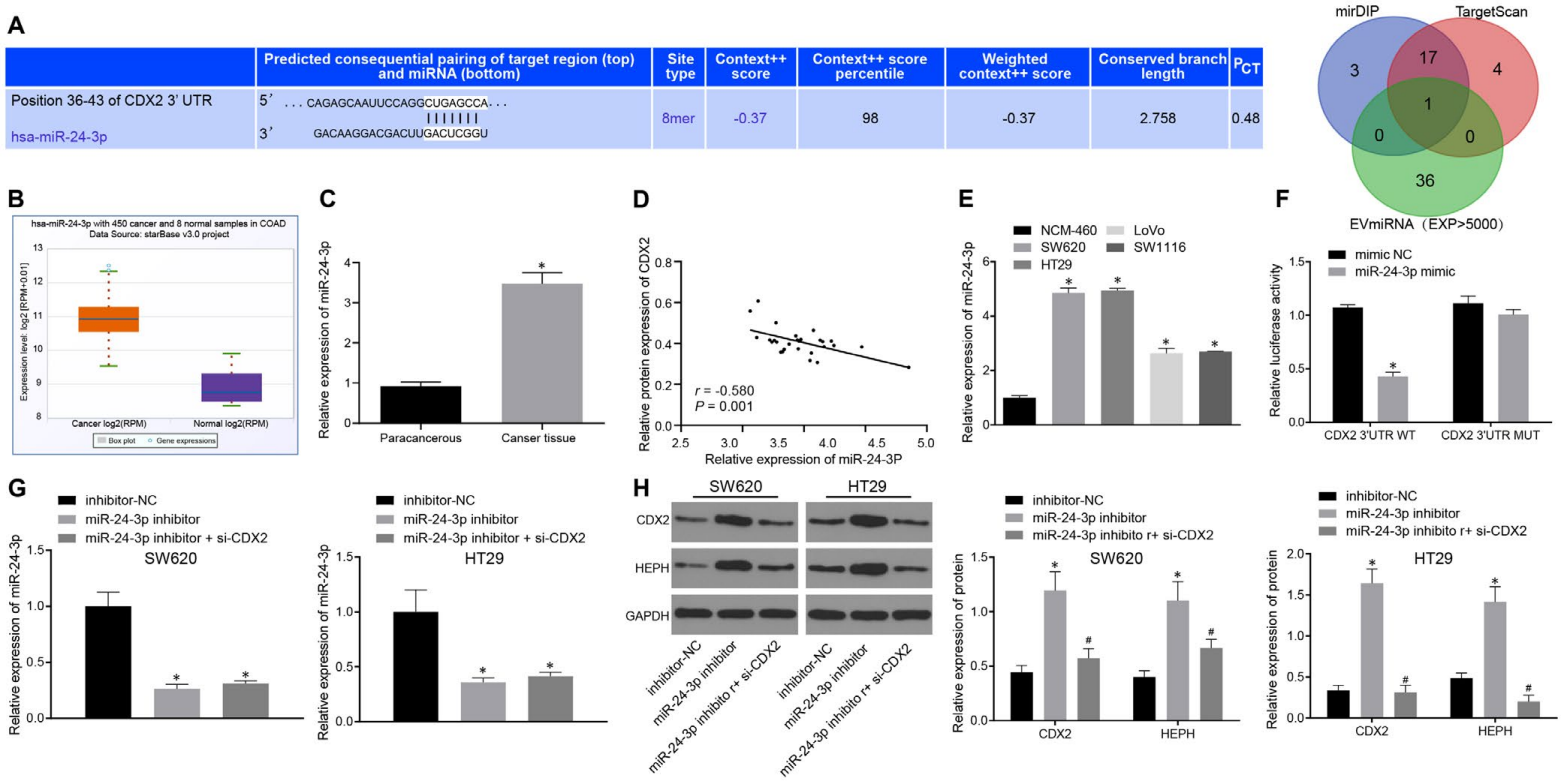
miR‐24‐3p is overexpressed in CC tissues and cells and CDX2 is a target gene of miR‐24‐3p. A, Prediction of upstream miRNA of CDX2. The 3 circles in the FIGURE represent the prediction results of the mirDIP and TargetScan database, and the miRNAs in the fibroblast microbubbles obtained on the EVmiRNA database (lower FIGURE). The binding site between miR‐24‐3p and CDX2 predicted on the TargetScan database (upper FIGURE). The middle part represents the intersection of the three sets of data. B, Expression of miR‐24‐3p in CC on TCGA. The red box plot represents the tumour samples, and the purple box plot represents the normal samples. C, The expression of miR‐24‐3p in CC tissues and adjacent tissues measured by RT‐qPCR normalized to U6. D, Spearman's correlation test of correlation between CDX2 mRNA expression and miR‐24‐3p expression in clinical samples of CC tissues. E, The expression of miR‐24‐3p in NCM‐460 cells and CC cells (SW620, HT29, LoVo and SW1116) examined by RT‐qPCR normalized to U6. F, The binding relationship between miR‐24‐3p and CDX2 assessed by dual‐luciferase reporter gene assay. G, The expression of miR‐24‐3p in cells after inhibition of miR‐24‐3p and CDX2 detected by RT‐qPCR normalized to U6. H, The protein expression of CDX2 and HEPH in cells after inhibition of miR‐24‐3p and CDX2 measured by Western blot analysis normalized to GAPDH. ^*^ *P* < .05 vs adjacent tissues, NCM‐460 cells or inhibitor‐NC‐transfected cells. ^#^ *P* < .05 vs miR‐24‐3p inhibitor–transfected cells. Measurement data were expressed as mean ± standard deviation. Data of clinical samples were compared using paired *t* test, and data between cells were compared by unpaired *t* test. Data between two groups were compared by paired *t* test, and comparisons among multiple groups were conducted by one‐way analysis of variance (ANOVA), followed by Bonferroni's post hoc test

The mirDIP and TargetScan were applied to predict the upstream regulatory miRNAs of CDX2. Meanwhile, miRNAs with the expression value of more than 5000 in fibroblast microbubbles were obtained in the EVmiRNA database. The intersection between the predicted results and EVmiRNA results showed that only one miRNA, miR‐24‐3p, was found in the intersection. The binding site between miR‐24‐3p and CDX2 was predicted on the TargetScan database (Figure 3A). miR‐24‐3p expression of CC in TCGA was searched, and the result revealed a high expression of miR‐24‐3p in CC (Figure 3B). miR‐24‐3p expression in CC tissues and adjacent tissues was detected by RT‐qPCR. It was suggested that miR‐24‐3p expression was prominently higher in CC tissues than in adjacent tissues (Figure 3C). Moreover, it was demonstrated that miR‐24‐3p expression was negatively correlated with the mRNA expression of CDX2 in clinical samples of CC (Figure 3D). miR‐24‐3p expression was determined in NCM‐460 cells and CC cells (SW620, HT29, LoVo and SW1116). The results displayed that miR‐24‐3p highly expressed in CC cells compared with NCM‐460 cells (Figure 3E), which was concurred with the trend in the clinical samples.

Subsequently, dual‐luciferase reporter gene assay was adopted to further verify whether CDX2 was a target gene of miR‐24‐3p. The results documented that the luciferase activity of CDX2‐WT in HEK‐293T cells transfected with miR‐24‐3p mimic was severely reduced. There was no significant change in luciferase activity of the CDX2‐MUT (Figure 3F). In addition, we successfully transfected miR‐24‐3p inhibitor and si‐CDX2 into SW620 and HT29 cells (Figure 3G), followed by measurement of CDX2 expression in SW620 and HT29 cells by Western blot analysis. The results presented that the protein expression of CDX2 and HEPH was significantly elevated in miR‐24‐3p inhibitor–transfected cells, which was rescued by si‐CDX2 (Figure 3H). The above results collectively demonstrated that miR‐24‐3p was overexpressed in CC tissues and cells, and that CDX2 was a target gene of miR‐24‐3p.

### 3.4 miR‐24‐3p promotes CC cell resistance to MTX through down‐regulation of CDX2

**Figure 4:**
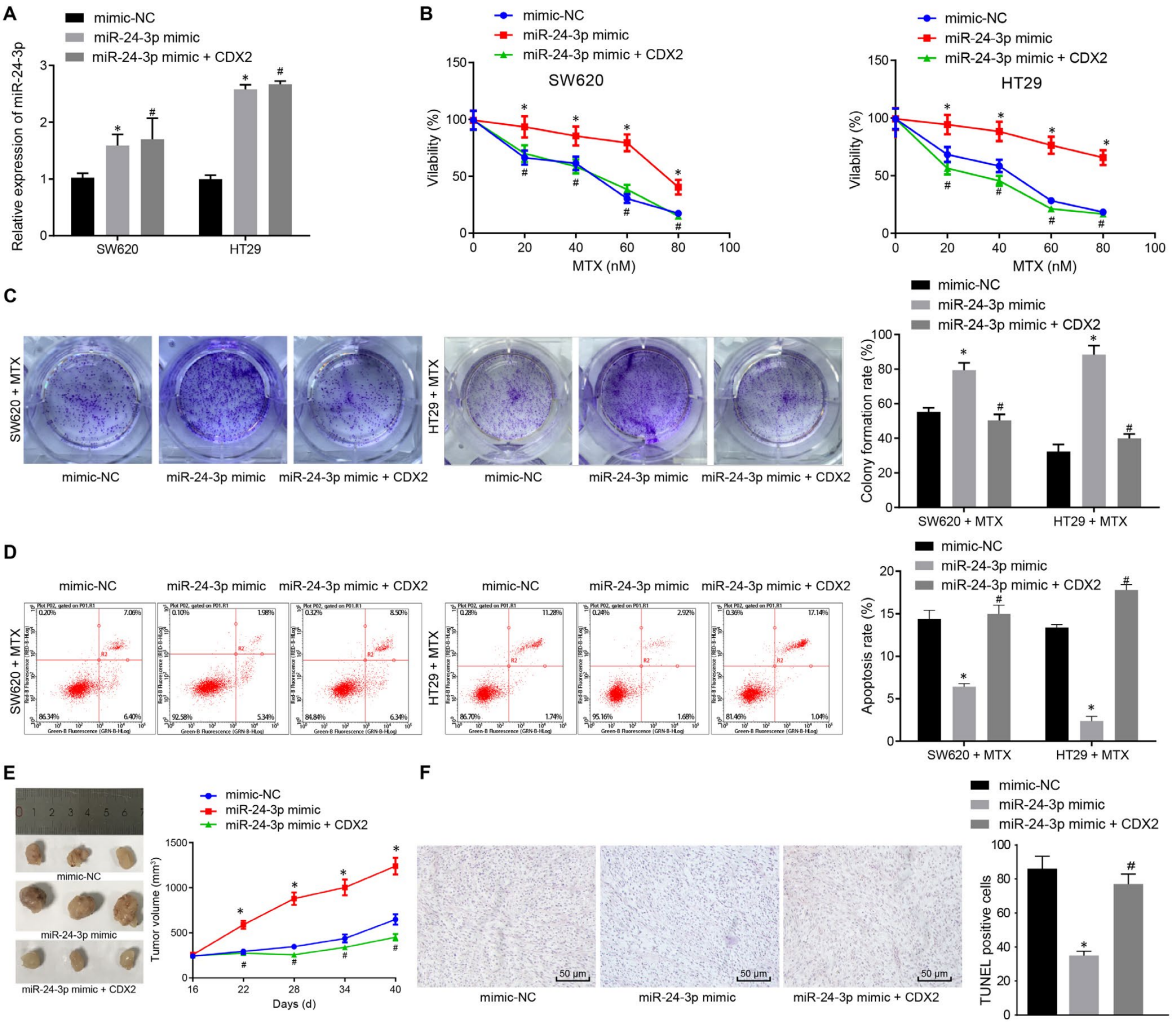
miR‐24‐3p enhances MTX resistance in CC *via* CDX2 down‐regulation. SW620 and HT29 cells were transfected with mimic NC, miR‐24‐3p mimic or miR‐24‐3p mimic + CDX2, followed by MTX treatment. A, The expression of miR‐24‐3p in SW620 and HT29 cells determined by RT‐qPCR normalized to U6. B, Cell viability of transfected SW620 and HT29 cells after 5 d of MTX treatment detected by CCK‐8 assay. C, Colony formation assessed by colony formation assay. D, Flow cytometry analysis of transfected SW620 and HT29 cell apoptosis. Mice were transfected with mimic NC, miR‐24‐3p mimic or miR‐24‐3p mimic + CDX2, followed by MTX treatment. E, Microscopic observation of tumour size and tumour volume (n = 10). F, TUNEL staining of apoptosis in tumour tissues (n = 10; ×200). ^*^ *P* < .05 vs cells or mice treated with mimic NC + MTX. ^#^ *P* < .05 vs cells or mice treated with miR‐24‐3p mimic + MTX. Measurement data were expressed as mean ± standard deviation. Comparisons among multiple groups were conducted by one‐way analysis of variance (ANOVA). Data at different time‐points were compared by repeated‐measures ANOVA, followed by Bonferroni's post hoc test

CDX2 was proven to be a downstream target gene of miR‐24‐3p. Next, we explored whether miR‐24‐3p regulated the resistance of CC cells to MTX *via* CDX2. SW620 and HT29 cells were transfected with miR‐24‐3p mimic and CDX2, followed by MTX treatment. CCK‐8 assay, colony formation assay and flow cytometry were conducted, the results of which showed that in MTX‐treated cells, viability and colony formation of SW620 and HT29 cells were markedly elevated, and apoptotic rate was reduced following transfection with miR‐24‐3p mimic, which was eliminated following the overexpression of CDX2 (Figure 4A‐D).

The results of tumour xenografts in nude mice revealed that size of subcutaneous tumours was noticeably enhanced in MTX‐treated mice treated with miR‐24‐3p mimic, which was abrogated after CDX2 overexpression (Figure 4E). In addition, MTX‐treated mice presented with reduced TUNEL‐positive apoptotic cells after treatment with miR‐24‐3p mimic, which was neutralized by up‐regulating CDX2 (Figure 4F). In summary, miR‐24‐3p enhanced the resistance to MTX by down‐regulating CDX2 in CC.

### 3.5 miR‐24‐3p is highly expressed in exosomes derived from CAFs

**Figure 5:**
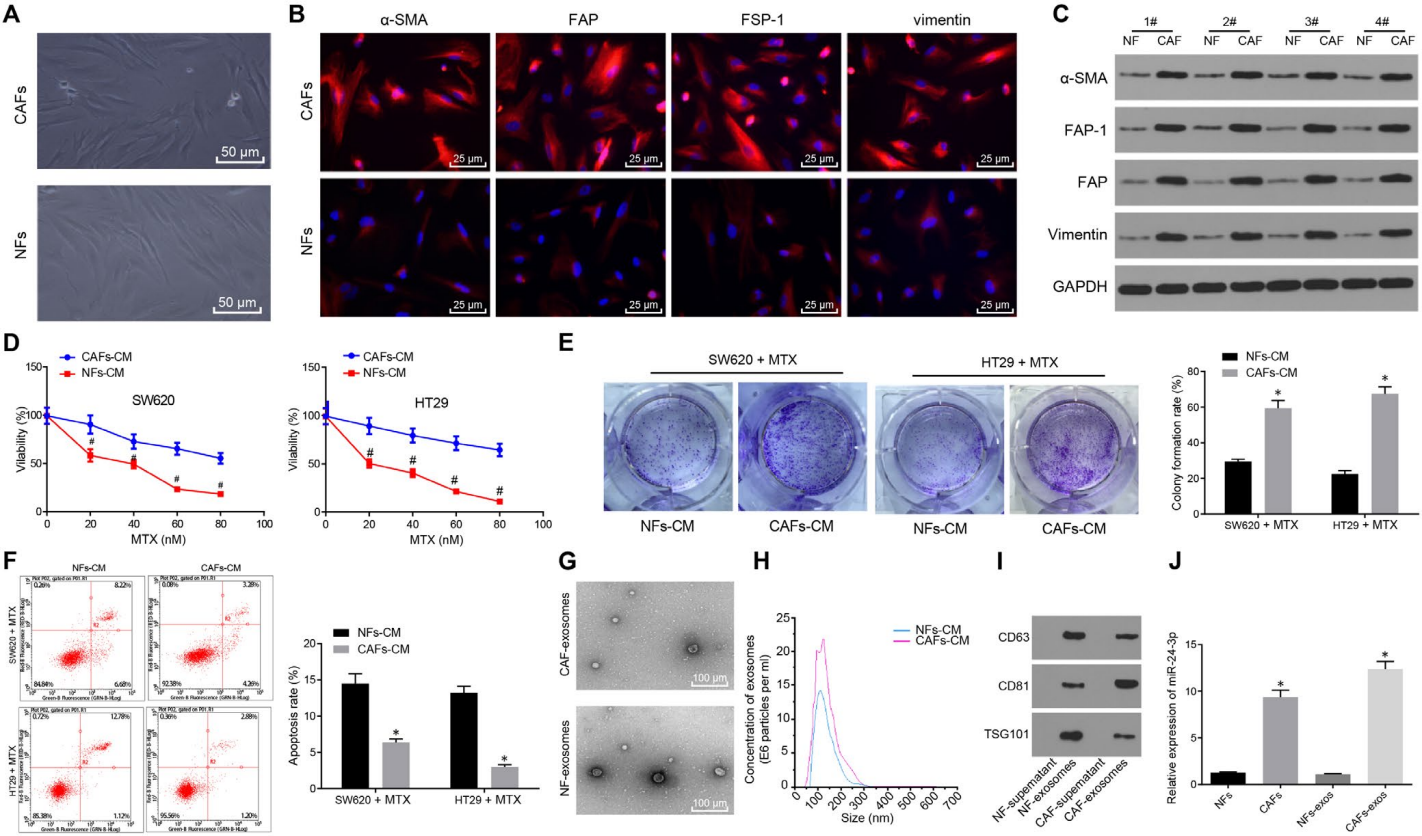
miR‐24‐3p shows high expression in exosomes derived from CAFs. A, Microscopic observation of primary CAFs and NFs from CC tissues and normal colonic mucosal tissues (×200). B‐C, Immunofluorescence (B, ×400) and immunoblotting (C) analysis of the expression of α‐SMA, FAP, FSP‐1 and vimentin in CAFs and NFs. D, Cell viability of SW620 and HT29 cells treated with CAFs‐CM or NFs‐CM for 48 h followed by 5 d of MTX treatment detected by CCK‐8 assay. E, Colony formation analysis after 48‐h treatment with CAFs‐CM or NFs‐CM. F, Apoptosis of SW620 and HT29 cells treated with CAFs‐CM or NFs‐CM for 48 h analysed by flow cytometry. G, Transmission electron micrographs of NFs‐exo and CAFs‐exo (scale bar: 100 nm). The white arrow pointed to the exosomes. H, Characterization of NFs‐exo and CAFs‐exo by nanosight analysis. I, The expression of the exosomal marker proteins (CD63, CD81 and TSG101) in NFs‐exo and CAFs‐exo determined by Western blot analysis. J, The expression of miR‐24‐3p in CAFs, NFs, CAFs‐exo and NFs‐exo examined by RT‐qPCR normalized to U6. ^*^ *P* < .05 vs NFs or NFs‐CM. Measurement data were expressed as mean ± standard deviation. Data between two groups were compared using unpaired *t* test. Comparisons among multiple groups were conducted by one‐way analysis of variance (ANOVA). Data at different time‐points were compared by repeated‐measures ANOVA, followed by Bonferroni's post hoc test. The cell experiment was repeated three times

Cancer‐associated fibroblasts and Normal fibroblasts were obtained from CC tissues and normal colonic mucosa. CAFs were positive for α‐SMA, FAP, FSP‐1 and vimentin, while these proteins were poorly expressed in NFs (Figure 5A‐C), which indicated that the isolated cells were CAFs and NFs. HT29 and SW620 cells were treated with CAFs‐conditioned medium (CAFs‐CM) or NFs‐CM. As revealed in Figure 5D‐F, compared with cells treated with NFs‐CM, cells treated with CAFs‐CM displayed stronger resistance to MTX. We hypothesized that CAFs might regulate CC cells by secreting exosomes. In order to test this hypothesis, exosomes were isolated from CAFs‐CM (CAFs‐exos) and NFs‐CM (NFs‐exos), respectively, by differential ultracentrifugation. Under the TEM, exosomes were in a discoid vesicle structure (Figure 5G). Nanosight analysis showed a mean particle size of 50‐100 nm, which was a typical feature of exosomes (Figure 5H). Furthermore, results of Western blot analysis revealed that the expression of the exosomal marker proteins (CD63, CD81 and TSG101) was positive (Figure 5I). miR‐24‐3p expression in CAFs, NFs, CAFs‐exo and NFs‐exo was examined by RT‐qPCR, which demonstrated that miR‐24‐3p was highly expressed in CAFs and CAFs‐exo (Figure 5J). The aforementioned results showed the presence of high miR‐24‐3p expression in CAF‐derived exosomes.

### 3.6 miR‐24‐3p can be transferred from CAFs to CC cells through exosomes

**Figure 6:**
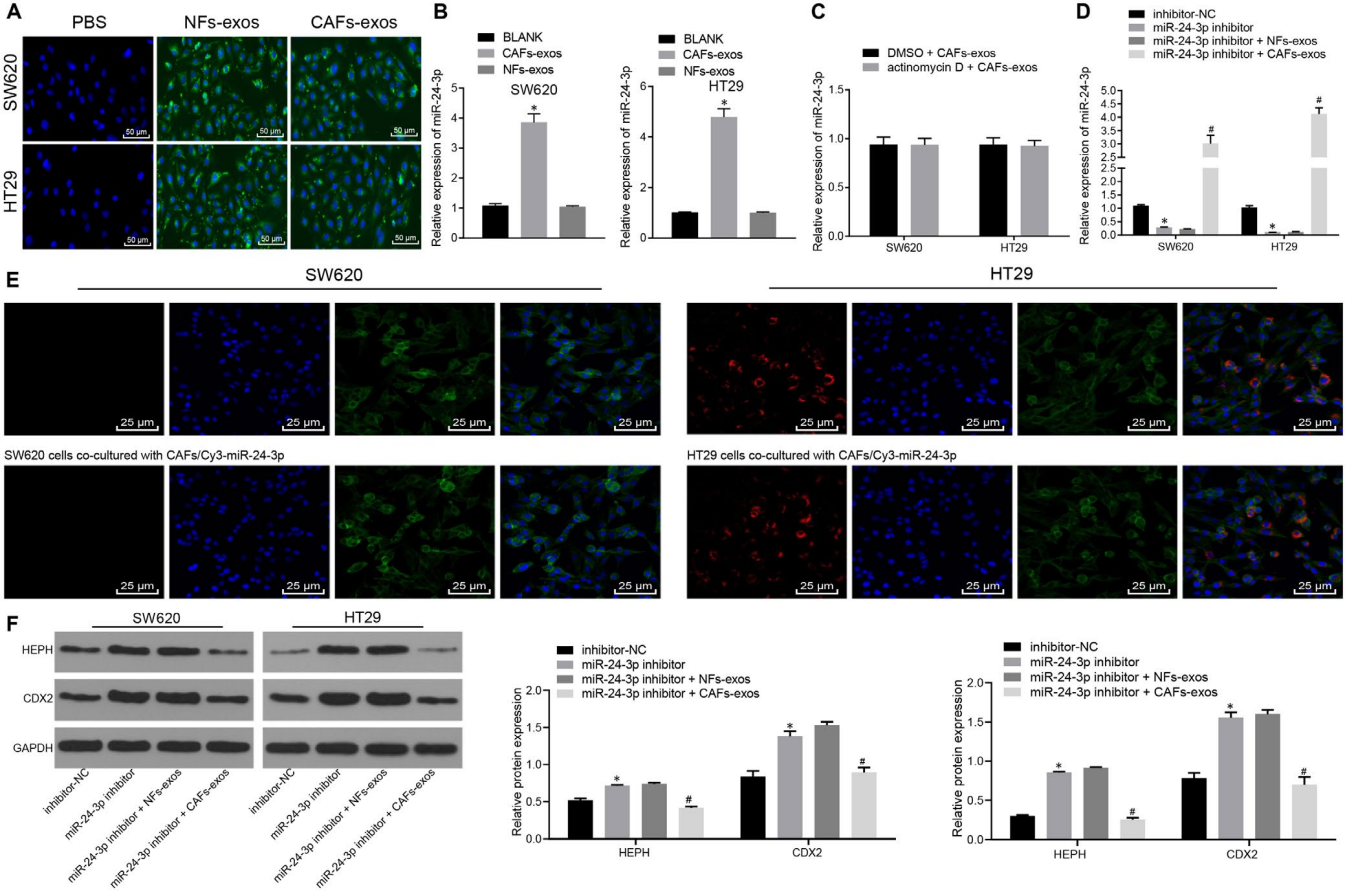
miR‐24‐3p can be transferred directly from CAFs to CC cells. A, The internalization of CAFs‐exo or NFs‐exo in HT29 and SW620 cells observed under a LSCM (×200). B, Expression of miR‐24‐3p in SW620 and HT29 cells co‐cultured with CAFs‐exo or NFs‐exo measured by RT‐qPCR normalized to U6. C, The expression of miR‐24‐3p in CC cells treated with CAFs‐exo after treatment of actinomycin D determined by RT‐qPCR normalized to U6. D, The expression of miR‐24‐3p in CC cells after treatment of miR‐24‐3p inhibitor and exosomes examined by RT‐qPCR normalized to U6. E, Images of HT29 cells or SW620 cells co‐cultured with CAFs transfected with Cy3‐labelled miR‐24‐3p observed under LSCM (×400). F, The protein expression of CDX2 and HEPH in CC cells after treatment of miR‐24‐3p inhibitor and exosomes analysed by Western blot analysis normalized to GAPDH. ^*^ *P* < .05 vs untreated HT29 cells or SW620 cells or HT29 or SW620 cells transfected with inhibitor‐NC. ^#^ *P* < .05 vs HT29 or SW620 cells transfected with miR‐24‐3p inhibitor + NFs‐exo. Measurement data were expressed as mean ± standard deviation. Data between two groups were compared using unpaired *t* test. Comparisons among multiple groups were conducted by one‐way analysis of variance (ANOVA), followed by Bonferroni's post hoc test. The cell experiment was repeated three times

CAFs‐exo or NFs‐exo were labelled with the green fluorescent dye PKH67 and added to CC culture medium to track whether these exosomes could be internalized by CC cells. Under the LSCM, CAFs‐exo– or NFs‐exo–treated HT29 and SW620 cells were observed to have green fluorescence signals, but no green fluorescence signal was observed in PBS‐treated cells (Figure 6A), indicating that PKH67‐labelled exosomes could be transferred into CC cells. To determine whether CAFs‐exo increased miR‐24‐3p expression in CC cells, miR‐24‐3p expression was measured in CC cells treated with CAFs‐exo or NFs‐exo. As described in Figure 6B, miR‐24‐3p expression was remarkably increased in SW620 and HT29 cells treated with CAFs‐exo, whereas miR‐24‐3p expression in SW620 and HT29 cells treated with NFs‐exo was not significantly elevated. The results of treatment of cells with actinomycin D revealed that the increase in miR‐24‐3p in CC cells was not the result of endogenous synthesis of miRNAs but the result of direct transfer of CAFs‐exo (Figure 6C). In order to further investigate whether the increase in miR‐24‐3p in CC cells was caused by direct transfer of CAFs, CC cells were first transfected with miR‐24‐3p inhibitor prior to incubation with CAFs‐exos or NFs‐exos. The results revealed significantly reduced miR‐24‐3p expression in CC cells following treatment with miR‐24‐3p inhibitor. However, the expression of miR‐24‐3p was significantly boosted in those cells after incubation with CAFs‐exo (Figure 6D). HT29 cells or SW620 cells were further co‐cultured with control CAFs or CAFs transfected with Cy3‐labelled miR‐24‐3p using the Transwell system. Interestingly, red fluorescence was observed in HT29 cells or SW620 cells co‐cultured with CAFs transfected with Cy3‐labelled miR‐24‐3p (Figure 6E). The results of Western blot analysis displayed that CDX2 and HEPH expression was sharply increased in CC cells transfected with miR‐24‐3p inhibitor and strikingly reduced after treatment with CAFs‐exo, but there was no significant difference in CDX2 and HEPH expression in cells treated with NFs‐exos (Figure 6F). Based on these findings, miR‐24‐3p could be directly transferred from CAFs to CC cells through exosomes, resulting in a significant decrease in CDX2 and HEPH expression in CC cells.

### 3.7 CAF‐derived exosome carrying miR‐24‐3p enhances CC cell resistance to MTX

**Figure 7:**
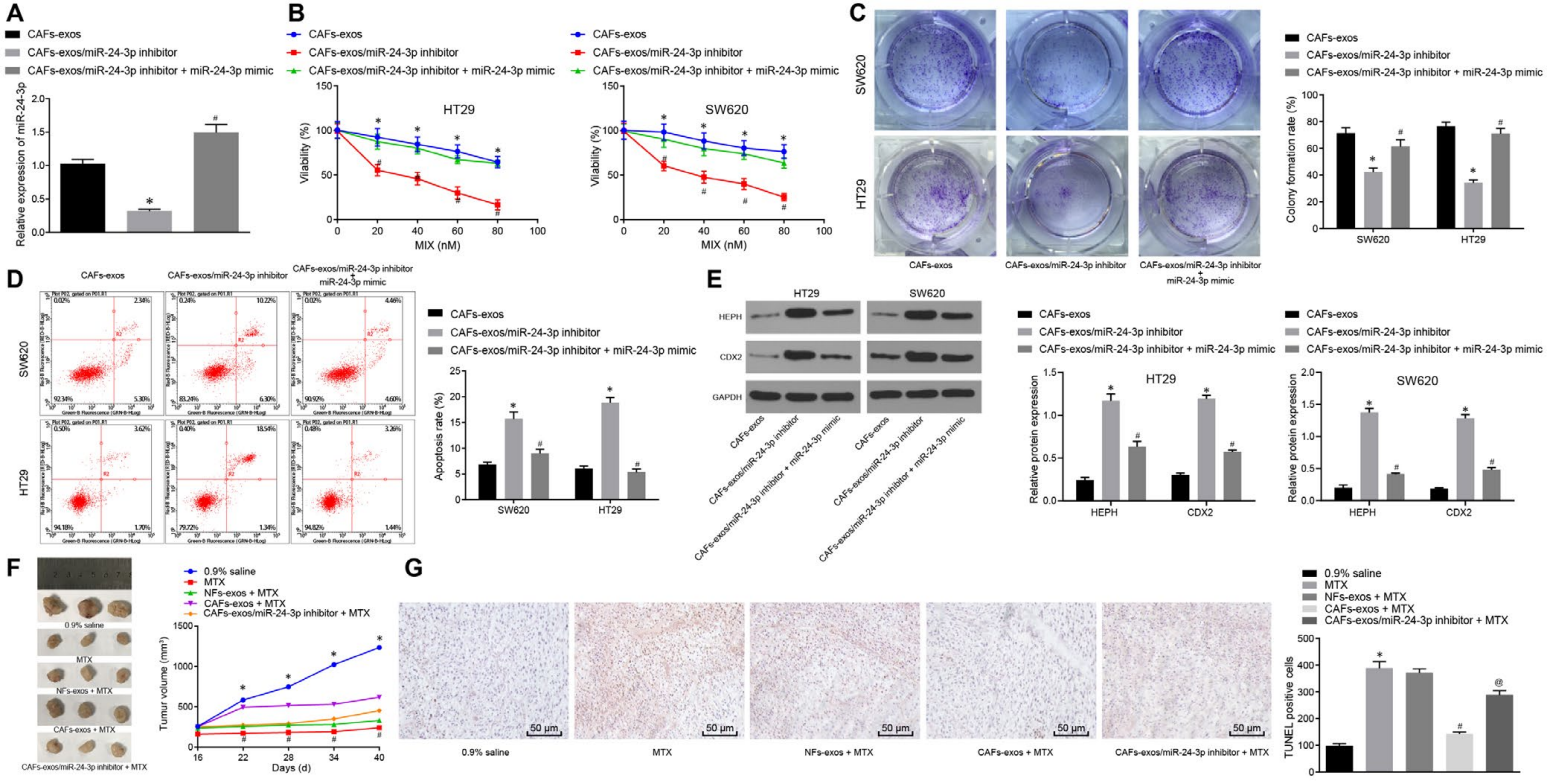
CAFs‐derived exosomal miR‐24‐3p promotes CC cell resistance to MTX. HT29 and SW620 cells were treated with CAFs‐exo, CAFs‐exo/miR‐24‐3p inhibitor or CAFs‐exo/miR‐24‐3p inhibitor + miR‐24‐3p mimic. A, The expression of miR‐24‐3p in exosomes measured by RT‐qPCR normalized to U6. B, Cell viability determined by CCK‐8 assay after cultured CC cells treated with MTX for 5 d. C, Colony formation analysis. D, Apoptosis analysed by flow cytometry. E, The protein expression of CDX2 and HEPH determined by Western blot analysis normalized to GAPDH. Mice were treated with 0.9% saline, MTX, NFs‐exo + MTX, CAFs‐exo + MTX or CAFs‐exo/miR‐24‐3p inhibitor + MTX (n = 10). F, The volume of the tumours. G, Apoptosis of the tumour tissue measured by TUNEL staining (n = 10; ×200). ^*^ *P* < .05 vs HT29 and SW620 cells treated with CAFs‐exo or mice injected with 0.9% saline. ^#^ *P* < .05 vs HT29 and SW620 cells treated with CAFs‐exo/miR‐24‐3p inhibitor or mice treated with MTX. ^@^ *P* < .05 *vs*. mice injected with CAFs‐exos + MTX. Measurement data were expressed as mean ± standard deviation. Comparisons among multiple groups were conducted by one‐way analysis of variance (ANOVA). Data at different time‐points were compared by repeated‐measures ANOVA, followed by Bonferroni's post hoc test. The cell experiment was repeated three times

The effect of CAF‐derived exosomal miR‐24‐3p on the treatment of CC by MTX in vitro and in vivo was explored. CAFs‐exo/miR‐24‐3p inhibitor significantly reduced the viability and colony formation of HT29 and SW620 cells and enhanced apoptosis compared with HT29 and SW620 cells treated with CAFs‐exo, which was reversed following treatment with miR‐24‐3p mimic (Figure 7A‐D). The results of Western blot analysis showed a significant increase in protein expression of CDX2 and HEPH in HT29 and SW620 cells following treatment of CAFs‐exo/miR‐24‐3p inhibitor, which was abolished when miR‐24‐3p mimic was re‐transfected into HT29 and SW620 cells (Figure 7E).

*In* in vivo experiments, the results displayed that the largest subcutaneous tumour was formed in mice by HT29 cells treated with 0.9% saline, while MTX‐treated HT29 cells formed the smallest subcutaneous tumour in nude mice. Under MTX treatment, HT29 cells treated with CAFs‐exo/miR‐24‐3p inhibitor formed smaller tumours than cells treated with CAFs‐exo but formed larger tumours than those treated with NFs‐exo (Figure 7F). In addition, apoptosis was reduced in tumour tissues of mice treated with CAFs‐exo compared with those treated with NFs‐exos. CAFs‐exo/miR‐24‐3p inhibitor induced apoptosis in tumour tissues of MTX‐treated mice (Figure 7G). Collectively, CAF‐derived exosomal miR‐24‐3p could enhance CC cell viability, inhibit apoptosis and promote tumour growth in vivo under MTX treatment.

## 4 DISCUSSION

While chemotherapy has been a successful therapeutic option for CC, the current challenge faced in these patients and main causes of death include drug resistance and metastasis.^25^ Thus, novel therapies tailored with the aid of molecular biomarkers are urgently needed to prevent resistance of CC cells to chemotherapy. Recent studies have highlighted the potential involvement of miRNAs in the chemotherapy resistance.^26^, ^27^ Reportedly, miR‐24‐3p regulated small‐cell lung cancer cell resistance to chemotherapy.^28^ Moreover, fibroblast*‐*derived microvesicles deliver miRNAs to tumour cells, mediating chemoresistance of tumour cells.^13^ Thus, the current study was designed to explore the effects of CAF‐derived exosomal miR‐24‐3p on the development of CC cell resistance to MTX and the potential downstream molecules. Collectively, our findings revealed that the transfer of exosomal miR‐24‐3p from CAFs into the CC cells resulted in the significant enhancement of MTX resistance through the down‐regulation of HEPH via CDX2.

Firstly, our result showed that low expression of HEPH and CDX2 was observed in CC clinical samples and MTX‐resistant CC cells, and that CDX2 positively regulated HEPH. A study conducted by Ma et al was consistent with the finding that CDX2 is down‐regulated in CC.^29^ Moreover, emerging evidence has demonstrated that CDX2 has tumour‐suppressing properties in CC by attenuating CC cell proliferation, migration invasion and metastasis.^30^, ^31^, ^32^ Similarly, the overexpression of CDX2 results in decreased viability and colony formation and boosted apoptosis in CC cells.^33^ Moreover, up‐regulation of CDX2 contributes to the suppression of proliferation and elevation of apoptosis of breast cancer epithelial cells.^34^ One intriguing discovery suggested that CDX2 is capable of up‐regulating the expression of HEPH in CC,^23^ which was in line with present findings. All of these results were consistent with our finding that CDX2 could elevate the expression of HEPH, promoting apoptosis and enhancing resistance of CC cells to MTX. Furthermore, corroborating studies have previously revealed that following its regulation with miRNAs, CDX2 plays a crucial role in disease progression. For instance, CDX2 down‐regulated by miR‐9 participates in biological progression of gastric cancer.^35^ Moreover, CDX2 was down‐regulated by miR‐2116‐3p in Barrett's oesophagus cells treated with omeprazole.^36^ Additionally, CDX2 is regulated by miR‐181d in hepatic cells.^37^ This evidence indirectly confirmed our results that miR‐24‐3p could target and down‐regulate CDX2, promoting MTX resistance in CC.

In addition, our result revealed a high expression of miR‐24‐3p in CC clinical samples and CC cells, which was also confirmed in breast cancer based on a previous study.^38^ Moreover, another study also validated the up‐regulation of miR‐24‐3p in CRC.^39^ In addition, our findings elucidated that miR‐24‐3p overexpression promoted cell proliferation and reduced cell apoptosis, thus inducing MTX resistance, which was supported by various studies. An example of such is the ability of miR‐24‐3p to accelerate cell migration and proliferation in lung cancer.^40^ Moreover, miR‐24‐3p has been proven to contribute to facilitation of cell proliferation, migration and invasion in bladder cancer.^41^ A study conducted by Liu et al revealed that miR‐24‐3p was one of the miRs, which could result in cell chemoresistance to cisplatin in ovarian cancer.^21^ Meanwhile, a prior study also reported that down‐regulated miR‐24‐3p resulted in the reduction of cell proliferation, colony formation and chemoresistance in head and neck squamous cell carcinoma.^42^

Another crucial finding from our study was that miR‐24‐3p can be transferred from CAFs to CC cells through exosomes and that CAF‐derived exosome carrying miR‐24‐3p leads to the enhancement of CC cell resistance to MTX. CAFs are mesenchymal cells in tumours, which are generated via the activation of resident mesenchymal cell populations and recruitment of bone marrow–derived mesenchymal stem cells and fibrocytes, with potential to promote CC progression.^43^ It is documented that secretion of exosomal miRNAs can communicate with CAFs with cancer cells.^44^ For instance, miR‐92a‐3p is confirmed to have a high expression in CAFs and CAF‐derived exosomal miR‐92a‐3p stimulates resistance of CRC cells to chemotherapy.^45^ Similarly, another study portrayed that miR‐21 can be transferred into tumour cells via CAF‐derived exosomes, promoting CRC development.^46^ These findings indirectly supported our results suggesting the presence of a high expression of miR‐24‐3p in CAFs and the transfer of miR‐24‐3p by exosomes derived from CAFs promoted MTX resistance in CC in vitro and in vivo.

**Figure 8:**
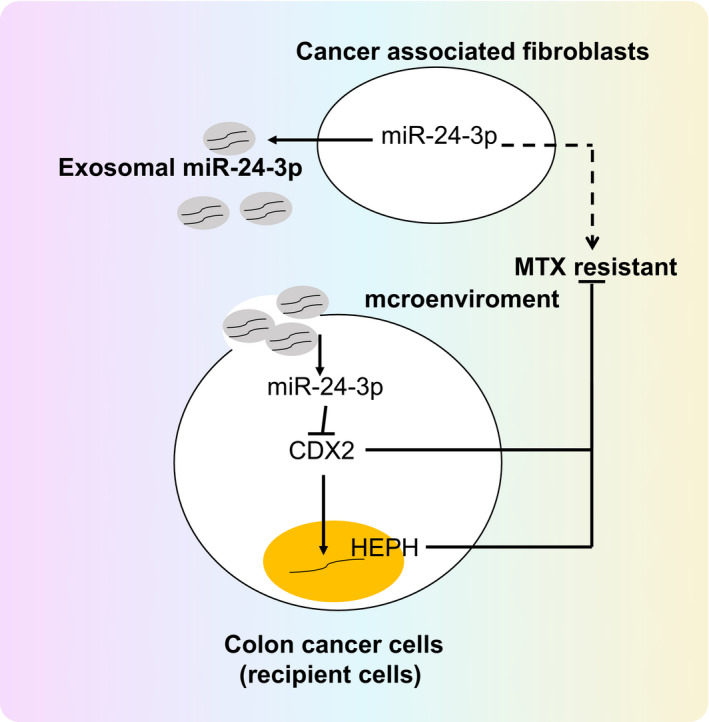
Schematic representation of function of CAF‐derived exosomal miR‐24‐3p in CC. Exosomal miR‐24‐3p derived from CAFs was released after endocytosis by CC cells. miR‐24‐3p could target CDX2, and CDX2 could positively regulate HEPH. miR‐24‐3p contributed to promotion of CC cell resistance to MTX, while CDX2 and HEPH inhibited CC cell resistance to MTX

In conclusion, the aforementioned findings elucidated that CAF‐derived exosomal miR‐24‐3p could function as an oncogene and was capable of enhancing the resistance of CC cells to MTX by activating the CDX2/HEPH axis (Figure 8), which provided a potential target to increase MTX sensitivity in clinical CC treatment. However, further studies are required to determine whether the therapeutic target is applicable to human beings.

## CONFLICT OF INTEREST

The authors declare that they have no competing interests.

## AUTHOR CONTRIBUTION

**Hong‐Wei Zhang:** Project administration (equal); Validation (equal); Writing‐review & editing (equal). **Yi Shi:** Project administration (equal); Validation (equal); Writing‐review & editing (equal). **Ji‐Bin Liu:** Conceptualization (equal); Investigation (equal); Writing‐original draft (equal). **Hui‐Min Wang:** Visualization (equal); Writing‐review & editing (equal). **Pei‐Yao Wang:** Visualization (equal); Writing‐review & editing (equal). **Zhi‐Jun Wu:** Data curation (equal); Formal analysis (equal). **Liu Li:** Data curation (equal); Formal analysis (equal). **Li‐Peng Gu:** Methodology (equal); Resources (equal). **Ping‐Sheng Cao:** Resources (equal); Software (equal). **Gao‐Ren Wang:** Investigation (equal); Writing‐original draft (equal). **Yu‐Shui Ma:** Conceptualization (equal); Investigation (equal); Writing‐original draft (equal). **Da Fu:** Data curation (equal); Formal analysis (equal); Supervision (equal).

## Supporting information

Figure S1

## Data Availability

The data that support the findings of this study are available from the corresponding author upon reasonable request.
